# Donepezil for dementia with Lewy bodies: a randomized, placebo-controlled, confirmatory phase III trial

**DOI:** 10.1186/s13195-014-0083-0

**Published:** 2015-02-03

**Authors:** Manabu Ikeda, Etsuro Mori, Kazutaka Matsuo, Masaki Nakagawa, Kenji Kosaka

**Affiliations:** Department of Neuropsychiatry, Faculty of Life Sciences, Kumamoto University, 1-1-1 Honjo, Chuo-ku, Kumamoto 860-8556 Japan; Department of Behavioral Neurology and Cognitive Neuroscience, Tohoku University Graduate School of Medicine, 2-1, Seiryo-machi, Aoba-ku, Sendai, Miyagi 980-8575 Japan; Eisai Product Creation Systems, Eisai Co. Ltd, 4-6-10 Koishikawa, Bunkyo-ku, Tokyo 112-8088 Japan; Department of Psychiatry, Yokohama City University School of Medicine, 3-9 Fukuura, Kanazawa-ku, Yokohama, Kanagawa 236-0004 Japan

## Abstract

**Introduction:**

The efficacy of a cholinesterase inhibitor, donepezil, in patients with dementia with Lewy bodies (DLB) was investigated to confirm the superiority over placebo in the 12-week, double-blind phase of this phase III study.

**Methods:**

Patients with probable DLB (*n* = 142) were randomly assigned to placebo or to 5 mg or 10 mg of donepezil administered once daily for 12 weeks. The co–primary endpoints were changes in cognitive function assessed using the Mini-Mental State Examination (MMSE) and behavioral and neuropsychiatric symptoms using the Neuropsychiatric Inventory (NPI-2: hallucinations and fluctuations). The superiority of each active group over placebo was determined with simultaneous statistical significance in both endpoints. Safety evaluations included adverse events (AEs) and the unified Parkinson's disease rating scale (UPDRS) part III.

**Results:**

The predefined superiority of donepezil to the placebo was not confirmed in either active group in the primary analysis. MMSE score significantly improved compared to placebo in the 10 mg group (10 mg: 2.2 ± 0.4, placebo: 0.6 ± 0.5 (mean ± standard error); *P* = 0.016). The change in MMSE score in the 5 mg group was not significant (1.4 ± 0.5 (mean ± standard error); *P* = 0.232). Although NPI-2 improved compared to baseline in the active groups, the differences from placebo were not significant. Most AEs were mild or moderate. Although the incidence of parkinsonism was slightly higher in the 10 mg group, the change in the UPDRS score was minimal and without a significant difference from the placebo group.

**Conclusions:**

The co–primary endpoints were not achieved in this trial. However, significant improvement in MMSE score was demonstrated with 10 mg, but not 5 mg, of donepezil. The evaluation of psychiatric symptoms might be affected by advanced education and instructions given to caregivers. Overall, donepezil was well tolerated in patients with DLB. With careful attention on gastrointestinal or parkinsonian symptoms, patients with DLB can safely benefit from treatment with donepezil.

**Trial registration:**

ClinicalTrials.gov Identifier: NCT01278407 (trial registration date: 14 January 2011)

**Electronic supplementary material:**

The online version of this article (doi:10.1186/s13195-014-0083-0) contains supplementary material, which is available to authorized users.

## Introduction

Dementia with Lewy bodies (DLB) is the second most common type of senile dementia following Alzheimer’s disease (AD) [1]. The core clinical features of DLB are fluctuating cognition, visual hallucinations and motor symptoms of parkinsonism, as well as cognitive impairment characterized by deficits in attention, executive function and visual perception [2]. Other features include neuropsychiatric symptoms such as delusions and depression, as well as autonomic dysfunction. Fluctuating cognition, hallucinations and delusions impose particular challenges and distress on both patients and caregivers. The motor and autonomic features have a further negative impact on activities of daily living and quality of life [3,4].

DLB is associated with a greater loss of cholinergic neurons in the nucleus basalis of Meynert and lower choline acetyltransferase (ChAT) activity than AD, but more postsynaptic muscarinic receptors in the cortex are preserved [5-7]. The cholinergic depletion correlates not only with the cognitive impairment but also with psychiatric symptoms such as hallucinations [8]. On the basis of these pathological features, it has been suggested that cholinesterase inhibitors (ChEIs) may be an effective treatment for DLB [9,10]. However, no ChEIs have been approved for DLB to date.

Previously, we examined the efficacy and safety of donepezil administered at 3, 5 and 10 mg for 12 weeks in patients with DLB in a placebo-controlled, double-blind exploratory study [11]. An open-label, long-term extension study was then conducted in patients who had completed the double-blind study to examine the safety and efficacy of donepezil at 5 mg for 52 weeks [12]. The double-blind study showed that donepezil at 5 mg or 10 mg per day significantly improved cognitive impairment, behavioral and psychiatric symptoms, global clinical symptoms and the caregiver burden compared to placebo. The long-term study showed that donepezil at 5 mg/day was well tolerated and that it sustained improvement in cognitive impairment and psychiatric symptoms over the course of 52 weeks.

The aim of the present phase III study, integrating a placebo-controlled, double-blind comparative study and an open-label long-term extension study, was to further evaluate the efficacy and to confirm the superiority of donepezil administration at 5 mg and 10 mg per day for 12 weeks over placebo, as well as to evaluate the safety and efficacy of long-term administration at 10 mg as well as 5 mg per day, in patients with DLB. This report describes the results of the placebo-controlled, double-blind, 12-week phase. Detailed results of the extension phase are reported elsewhere [13].

## Methods

### Patients

Patients diagnosed as probable DLB according to the consensus diagnostic criteria [2] were recruited from 72 psychiatric or neurological specialty centers throughout Japan from February 2011 to March 2012. Eligible patients were outpatients aged ≥50 years with mild to moderate or severe dementia (10 to 26 on the Mini-Mental State Examination (MMSE) and Clinical Dementia Rating ≥0.5) and behavioral and psychiatric symptoms (Neuropsychiatric Inventory-plus (NPI-plus) ≥8 and NPI (NPI-2) ≥1). The NPI-plus consisted of 12 items: the original 10 items, sleep [14,15] and cognitive fluctuation, which is reported as the Cognitive Fluctuation Inventory [16,17] (see Additional file 1). The NPI-2 consisted of hallucinations and cognitive fluctuation [11]. The caregivers of the eligible patients had to routinely stay with them at least 3 days per week and 4 hours per day, provide information for this study, assist with the compliance with treatment and escort them to required visits.

The exclusion criteria included Parkinson’s disease that was diagnosed at least 1 year prior to the onset of dementia; focal vascular lesions visualized on magnetic resonance imaging or computed tomographic scans that might cause cognitive impairment; other neurological or psychiatric diseases; clinically significant systemic disease; complications or a history of severe gastrointestinal ulcer, severe asthma or obstructive pulmonary disease; systolic hypotension (<90 mmHg); bradycardia (<50 m^−1^); sick sinus syndrome; atrial or atrioventricular conduction block; QT interval prolongation (≥450 ms); hypersensitivity to donepezil or piperidine derivatives; severe parkinsonism (Hoehn and Yahr stage IV or above) [18]; and treatment with ChEIs or any investigational drug within 3 months prior to screening. ChEIs, antipsychotics and antiparkinson drugs other than l-dopa or dopamine agonists were not allowed during the study.

### Randomization and masking

**Figure 1:**
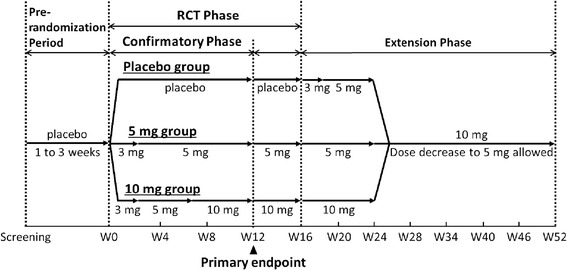
**Study flow.** RCT, Randomized placebo-controlled trial.

This study consisted of two phases: a 16-week, double-blind randomized control (RCT) phase and a subsequent 36-week, open-label extension phase. Treatment with donepezil lasted up to 52 weeks in total. The RCT phase, which was preceded by a 2-week (1 to 3 weeks) prerandomization period, entailed the 12-week confirmatory phase (Figure 1). In this article, we report the results of the confirmatory phase. All patients were given placebo tablets during the prerandomization period, after which the patients were assigned in a 1:1:1 ratio to placebo or 5 mg or 10 mg of donepezil in the RCT phase. Randomization was performed centrally according to a dynamic allocation, adjusting for MMSE and NPI-2 scores at screening. A member of the research staff who was in charge of randomization and who was independent of all the parties concerned with the study securely kept the randomization list with limited access only in an emergency. No other members of the research staff, including the physicians, nurses and study institution staff were aware of the treatment assignment, nor were any of the participants.

Patients received two study drug tablets, which were composed of a combination of 3 mg, 5 mg, or the matched placebo tablets with the same physical appearance, once daily in the morning. The dosage was titrated at the beginning. Treatment began with 3 mg for 2 weeks, and then the dose was increased to 5 mg. Thereafter, the dose was increased to 10 mg at week 6 only in the 10 mg group. The dose was escalated after patient safety was confirmed. Dose reduction was not permitted in the RCT phase.

### Procedures

In the confirmatory phase, efficacy was assessed at baseline and at weeks 4, 8 and 12. Co–primary endpoints were cognitive function assessed using the MMSE [19] and behavioral and neuropsychiatric symptoms assessed using the NPI-2 [11], both at week 12. NPI-2 was calculated as the sum of the scores for hallucinations and cognitive fluctuation, which corresponded to two of the core features of DLB in the consensus criteria. The original NPI-10 (delusions, hallucinations, agitation/aggression, dysphoria, anxiety, euphoria, apathy, disinhibition, irritability/lability and aberrant motor behavior) was set as a secondary endpoint.

Caregiver burden was assessed using the Zarit Caregiver Burden Interview (ZBI) [20], which evaluates the physical, psychological and social consequences of care activities. The ZBI contains 22 items scored from 0 (best) to 4 (worst), from which a total score of 0 to 88 is calculated.

Safety was assessed based on adverse events (AEs), vital signs, electrocardiograms and laboratory tests. All AEs were classified and coded according to Medical Dictionary for Regulatory Activities (“MedDRA”) terms. Gastrointestinal symptoms, parkinsonian symptoms, psychiatric symptoms and arrhythmia were assessed as AEs of interest. Motor function was assessed as a safety measure using the Unified Parkinson’s Disease Rating Scale (UPDRS) part III [21] at baseline and week 12.

Written informed consent was obtained from the patients (if at all possible) and their primary caregiving family members before initiating the study procedures. The study was conducted in accordance with the principles of the Declaration of Helsinki. The protocol was approved by the institutional review board at each center (Additional file 2).

### Statistical analyses

In a sample size calculation, the mean changes in MMSE score were estimated to be −0.4, 2.0 and 2.0 with standard deviation (SD) of 3.3, and the mean changes in NPI-2 score were estimated to be 1.1, −3.3 and −4.6 with SD of 5.2 in the placebo, 5 mg and 10 mg groups, respectively, according to the results of the previous double-blind study. The Bonferroni-corrected significance level was set at one-sided 1.25%. Detecting the significant difference, predefined to be determined only with statistical significance in both MMSE and NPI-2 results, with at least 80% statistical power between the placebo and 5 mg groups required at least 126 patients (42 per group) (statistical power of 80.7%). The number was expected to provide power of 85.4% to detect a significant difference between the placebo and 10 mg groups. Given that 10% of the patients were excluded from the full analysis set (FAS), the target number of patients in this study was set at 141.

Efficacy was analyzed in the FAS and the per-protocol set (PPS). Analysis using the FAS was positioned for primary analysis. Mean changes from the baseline in each outcome measure were compared between each active group and placebo by analysis of covariance (ANCOVA) with baseline values as covariates. Only the statistical significance in both MMSE and NPI-2 between the placebo group and each active group could determine the superiority of the active drug over placebo. The significance level was adjusted for multiplicity using the Hochberg method. In addition, MMSE improvement was evaluated by responder rate, defined as the proportion of patients with ≥3-point improvement.

The safety analysis set comprised all patients who received at least one dose and had a postbaseline safety assessment. The incidence of AEs was summarized by group. For laboratory parameters and vital signs, descriptive statistics and frequency distributions were calculated. UPDRS part III scores were compared between each active group and the placebo group using ANCOVA with baseline values as covariates.

All analyses were carried out using SAS versions 9.1 and 9.2 software (SAS Institute, Cary, NC, USA).

## Results

### Patients

**Figure 2:**
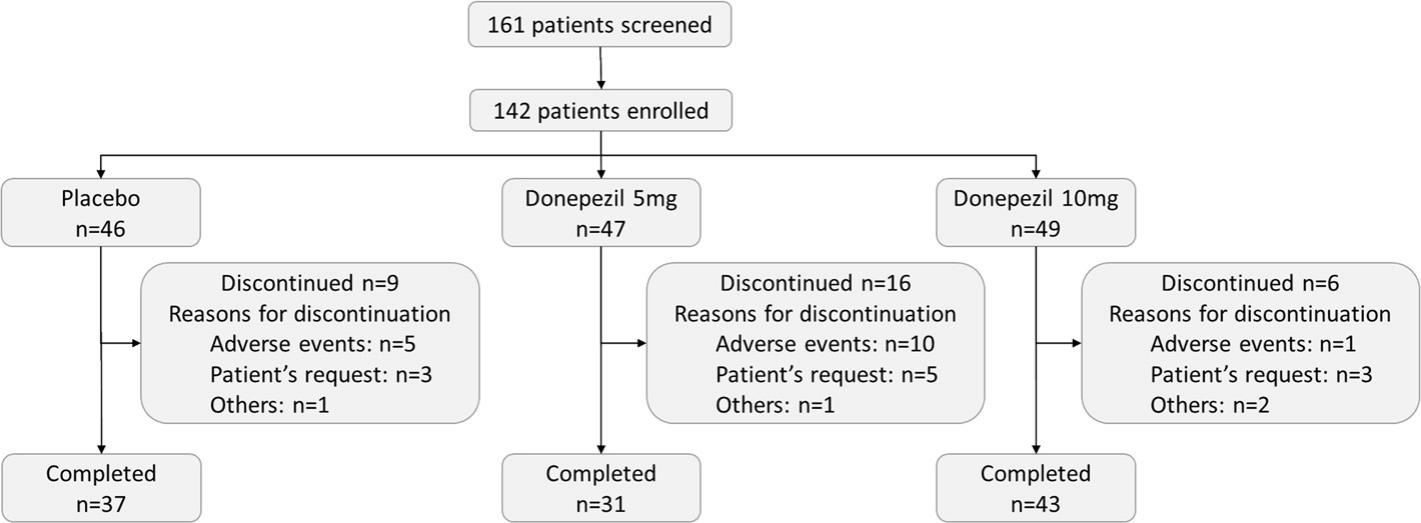
**Patient disposition in the confirmatory phase.**

Of 161 patients enrolled in the prerandomization period, 142 were enrolled in the RCT phase and randomized to the placebo, 5 mg and 10 mg groups (46, 47 and 49 patients, respectively) (Figure 2). Of these patients, 138 were included in the FAS (44, 45 and 49 patients in the placebo, 5 mg and 10 mg groups, respectively). Four patients (two patients each in the placebo and 5 mg groups) were excluded because of a lack of evaluable efficacy data (three patients) and doubtful diagnosis of probable DLB (one patient). Excluding 19 patients from the FAS, 119 patients (40, 34 and 45 patients in the placebo, 5 mg and 10 mg groups, respectively) constituted the PPS. The reasons for the 19 exclusions were discontinuation within <8 weeks, compliance rate <75% or lack of efficacy data due to a change in the evaluator.

Thirty-one patients discontinued (9, 16 and 6 patients in the placebo, 5 mg and 10 mg groups, respectively) with more discontinuations in the 5 mg group than in the 10 mg group. The total discontinuations in the active groups comprised 22 (22.9%) of the 96 patients, which was similar to the placebo group (19.6%).

Demographic and baseline characteristics of the FAS are summarized in Table 1. There were no characteristic differences among the three groups. Females accounted for 58.0%. The mean age was 77.9 (range, 57 to 95) years. All but two patients were 65 years of age or older. Dementia medication had previously been used by only 5.8% of the patients. The mean MMSE score at baseline was 20.4 points.

**Table 1:** **Patient demographics and baseline characteristics** ^**a**^ **(FAS,** ***N*** **= 138)**

		**Donepezil**		
	**Placebo**	**5 mg**	**10 mg**	**Overall**
**Characteristics**	**(** ***n*** **= 44)**	**(** ***n*** **= 45)**	**(** ***n*** **= 49)**	**(** ***n*** **= 138)**
Sex, *n* (%)				
Male	17 (38.6)	20 (44.4)	21 (42.9)	58 (42.0)
Female	27 (61.4)	25 (55.6)	28 (57.1)	80 (58.0)
Age, yr	77.2 ± 6.1	78.8 ± 5.1	77.7 ± 6.8	77.9 ± 6.1
Weight, kg	50.15 ± 10.75	50.68 ± 9.24	51.72 ± 9.89	50.88 ± 9.92
Duration of dementia, yr	2.0 ± 2.3	2.7 ± 1.8	2.3 ± 1.9	2.3 ± 2.0
History of anti-dementia medication, *n* (%)				
Yes	1 (2.3)	3 (6.7)	4 (8.2)	8 (5.8)
No	43 (97.7)	42 (93.3)	45 (91.8)	130 (94.2)
Cognitive fluctuation, *n* (%)				
Yes	40 (90.9)	41 (91.1)	46 (93.9)	127 (92.0)
No	4 (9.1)	4 (8.9)	3 (6.1)	11 (8.0)
Visual hallucinations, *n* (%)				
Yes	42 (95.5)	39 (86.7)	39 (79.6)	120 (87.0)
No	2 (4.5)	6 (13.3)	10 (20.4)	18 (13.0)
Parkinsonism, *n* (%)				
Yes	38 (86.4)	39 (86.7)	44 (89.8)	121 (87.7)
No	6 (13.6)	6 (13.3)	5 (10.2)	17 (12.3)
Hoehn and Yahr stage, *n* (%)				
I	4 (9.1)	8 (17.8)	7 (14.3)	19 (13.8)
II	15 (34.1)	17 (37.8)	19 (38.8)	51 (37.0)
III	19 (43.2)	14 (31.1)	18 (36.7)	51 (37.0)
MMSE score	20.3 ± 4.2	20.6 ± 4.1	20.3 ± 4.8	20.4 ± 4.3
NPI-2 score	6.9 ± 4.5	6.9 ± 4.5	7.3 ± 4.7	7.1 ± 4.5
NPI-10 score	20.5 ± 15.0	18.9 ± 15.3	16.6 ± 11.7	18.6 ± 14.0
ZBI score	28.4 ± 16.2	28.3 ± 18.5	31.4 ± 17.8	29.4 ± 17.4

^a^FAS, Full analysis set; MMSE, Mini-Mental State Examination; NPI, Neuropsychiatric Inventory; ZBI, Zarit Caregiver Burden Interview. Values are expressed as mean ± SD, unless otherwise specified.

### Co–primary endpoints (MMSE and NPI-2 scores)

Changes in the co–primary endpoints (MMSE and NPI-2 scores) from baseline are shown in Table 2. Primary analysis did not confirm the predefined superiority of either active group to the placebo group.

**Table 2:** **Co–primary endpoints (MMSE and NPI-2 scores) and changes from baseline (FAS LOCF)** ^**a**^

	**Groups**	***n***	**Baseline (week 0) score**	**Week 12 (LOCF) change**	
			**Mean ± SD**	**Mean ± SD**	***P-*** **value** ^**b**^
MMSE^c^	Placebo	44	20.3 ± 4.2	0.6 ± 3.0	
	5 mg	45^d^	20.6 ± 4.1	1.4 ± 3.4	0.232
	10 mg	49	20.3 ± 4.8	2.2 ± 2.9	0.016
NPI-2^e^	Placebo	44	6.9 ± 4.5	−2.0 ± 4.2	
	5 mg	45	6.9 ± 4.5	−1.7 ± 4.3	0.661
	10 mg	49	7.3 ± 4.7	−2.9 ± 4.7	0.391

^a^FAS, Full analysis set; LOCF, Last observation carried forward; MMSE, Mini-Mental State Examination; NPI, Neuropsychiatric Inventory. ^b^Analysis of covariance with treatment groups as factors and baseline values as covariates. Significance was defined as *P* < 0.05. ^c^Positive value of the MMSE change indicates an improvement in cognitive function. ^d^The number of patients at week 12 (LOCF) was 43. ^e^A negative value of the NPI-2 change indicates an improvement in behavioral and neuropsychiatric symptoms.

### Cognitive function

Mean changes from baseline in MMSE in the FAS and PPS are shown in Table 3. In the FAS, the mean change from baseline in the MMSE scores at week 12 (last observation carried forward (LOCF)) was higher in each active group (mean ± standard error (SE): 1.4 ± 0.5 and 2.2 ± 0.4 in the 5 mg and 10 mg groups, respectively) than in the placebo group (mean ± SE: 0.6 ± 0.5). Improvement in the 10 mg group was significant compared to that in the placebo group (mean difference from placebo = 1.6; *P* = 0.016), but that in the 5 mg group was not (mean difference from the placebo = 0.8, *P* = 0.232). PPS analysis yielded a significant improvement in both active groups (5 mg: *P* = 0.025, 10 mg: *P* = 0.004). The responder rate (MMSE score change ≥3) was higher in each active group than in the placebo group (29.5%, 41.9% and 42.9% in the placebo, 5 mg and 10 mg groups, respectively).

**Table 3:** **Mean changes in Mini-Mental State Examination (MMSE) scores from baseline (LOCF)** ^**a**^

	**Group**	***n***	**Mean ± SE** ^**b**^	**Difference from placebo group** ^**b**^ **(95% CI)**	***P-*** **value**
FAS-LOCF	Placebo	44	0.6 ± 0.5	–	–
	5 mg	43	1.4 ± 0.5	0.8 (−0.5, 2.1)	0.232
	10 mg	49	2.2 ± 0.4	1.6 (0.3, 2.8)	0.016^c^
FAS-OC	Placebo	37	1.0 ± 0.5	–	–
	5 mg	32	2.2 ± 0.5	1.2 (−0.2, 2.7)	0.083
	10 mg	43	2.6 ± 0.4	1.6 (0.3, 2.9)	0.014^c^
PPS-LOCF	Placebo	40	0.5 ± 0.5	–	–
	5 mg	34	2.1 ± 0.5	1.6 (0.2, 2.9)	0.025^c^
	10 mg	45	2.4 ± 0.4	1.9 (0.6, 3.2)	0.004^c^

^a^CI, Confidence interval; FAS, Full analysis set; LOCF, Last observation carried forward; MMSE, Mini-Mental State Examination; OC, Observed case; PPS, Per-protocol set. ^b^Least squares mean from analysis of covariance with treatment groups as factors and baseline values as covariates. A positive value of the MMSE change indicates improvement in cognitive function. ^c^ *P* <0.05.

### Behavioral and neuropsychiatric symptoms

Changes from baseline in NPI-2 and NPI-10 scores are shown in Table 4. The changes in NPI-2 scores in both active groups were not significantly different from that in the placebo group. In the active group, NPI-2 improved at week 12 (LOCF) (mean ± SE: −1.8 ± 0.6 and −2.8 ± 0.5 in the 5 mg and 10 mg groups, respectively). However, the placebo group also showed improvement of −2.1 ± 0.6 (mean ± SE). The NPI-10 score improved at week 12 (LOCF) in each active group by −3.3 ± 1.4 and −5.5 ± 1.4 (mean ± SE) in the 5 mg and 10 mg groups, respectively, and also in the placebo group, by −6.4 ± 1.5. There was no significant difference between either of the active groups and the placebo group.

**Table 4:** **Change in NPI from baseline (FAS-LOCF)** ^**a**^

	**Group**	***n***	**Mean ± SE** ^**b**^	**Difference from placebo group** ^**b**^ **(95% CI)**	***P-*** **value**
NPI-2	Placebo	44	−2.1 ± 0.6	–	–
	5 mg	45	−1.8 ± 0.6	0.4 (−1.3, 2.0)	0.661
	10 mg	49	−2.8 ± 0.5	−0.7 (−2.3, 0.9)	0.391
NPI-10	Placebo	44	−6.4 ± 1.5	–	–
	5 mg	45	−3.3 ± 1.4	3.0 (−1.0, 7.1)	0.143
	10 mg	49	−5.5 ± 1.4	0.9 (−3.1, 4.9)	0.660

^a^FAS, Full analysis set; LOCF, Last observation carried forward; NPI, Neuropsychiatric Inventory. ^b^Least squares mean from analysis of covariance with treatment groups as factors and baseline values as covariates. A negative value of the NPI change indicates an improvement in behavioral and neuropsychiatric symptoms.

### Caregiver burden

ZBI score at week 12 (LOCF) was almost unchanged from the baseline in the placebo group (mean ± SE: −0.1 ± 1.8). In both the 5 mg and 10 mg groups, the score improved by −5.0 ± 1.8 and −0.8 ± 1.7 points (mean ± SE), respectively, but without a significant difference from the placebo group. Subgroup analysis yielded a stronger trend of the ZBI improvement in a group of caregivers who lived with the patient, and a significant difference between the 5 mg group and the placebo group (FAS-LOCF: *P* = 0.017).

### Safety

The incidence of AEs and treatment-related AEs did not differ substantially among the groups (AEs: 67.4% (31 of 46), 63.8% (30 of 47) and 69.4% (34 of 49); treatment-related AEs: 23.9% (11 of 46), 25.5% (12 of 47) and 28.6% (14 of 49) in the placebo, 5 mg and 10 mg groups, respectively). The incidence of severe or serious AEs in either of the active groups (severe AEs: 8.5% (4 of 47) and 0% (0 of 49); serious AEs: 8.5% (4 of 47) and 2.0% (1 of 49) in the 5 mg and 10 mg groups, respectively) did not substantially exceed those in the placebo group (severe AEs: 6.5% (3 of 46); serious AEs: 10.9% (5 of 46)). The incidence of the AEs that led to discontinuation was higher in the 5 mg group (21.3% (10 of 47)), but lower in the 10 mg group (4.1% (2 of 49)), than in the placebo group (10.9% (5 of 46)).

AEs with an incidence ≥5% in any treatment group are shown in Table 5. Major AEs with a higher incidence in either of the active groups than in the placebo group were parkinsonism (4.3% (2 of 46), 4.3% (2 of 47) and 8.2% (4 of 49), in the placebo, 5 mg and 10 mg groups, respectively, provided in the same order hereinafter), decreased appetite (2.2% (1 of 46), 6.4% (3 of 47), and 4.1% (2 of 49)) and nausea (2.2% (1 of 46), 6.4% (3 of 47) and 2.0% (1 of 49)). The incidence of contusion in the active groups (0.0% (0 of 47) and 2.0% (1 of 49) in the 5 mg and 10 mg groups, respectively) was low compared to the placebo group (8.7% (4 of 46)).

**Table 5:** **Adverse events with an incidence of more than 5% in any treatment groups** ^**a**^

**AE**	**Placebo group (** ***n*** **= 46)**		**5 mg group (** ***n*** **= 47)**		**10 mg group (** ***n*** **= 49)**	
	**AEs**	**Treatment-related AEs** ^**b**^	**AEs**	**Treatment-related AEs** ^**b**^	**AEs**	**Treatment-related AEs** ^**b**^
Total incidence	31 (67.4)	11 (23.9)	30 (63.8)	12 (25.5)	34 (69.4)	14 (28.6)
Nausea	1 (2.2)	1 (2.2)	3 (6.4)	2 (4.3)	1 (2.0)	1 (2.0)
Pyrexia	0	0	0	0	3 (6.1)	0
Nasopharyngitis	7 (15.2)	0	4 (8.5)	0	2 (4.1)	0
Contusion	4 (8.7)	0	0	0	1 (2.0)	0
Decreased appetite	1 (2.2)	1 (2.2)	3 (6.4)	1 (2.1)	2 (4.1)	2 (4.1)
Parkinsonism	2 (4.3)	2 (4.3)	2 (4.3)	2 (4.3)	4 (8.2)	4 (8.2)
Pollakiuria	0	0	3 (6.4)	3 (6.4)	0	0

^a^AE, Adverse event. Incidence shown as number and percentage. ^b^AEs for which a causal relationship with the study drug was considered possible or probable.

The incidence of gastrointestinal events in the 5 mg group was higher than in the placebo group, but that in the 10 mg group was similar to the placebo group (13.0% (6 of 46), 21.3% (10 of 47) and 14.3% (7 of 49)). Decreased appetite and nausea were both observed in >5% of patients in the 5 mg group, but the incidence of no gastrointestinal events in the 10 mg group reached 5%. All gastrointestinal events were mild or moderate in severity. When analyzed in 14-day intervals from the baseline, the incidence in the 10 mg group at the interval of days 43 to 56, the first interval following the dose increase from 5 to 10 mg at week 6, was the highest among the periods and the groups (8.3%).

As parkinsonian AEs, only parkinsonism was reported, and its incidence was slightly higher in the 10 mg group than in the placebo and 5 mg groups (4.3% (2 of 46), 4.3% (2 of 47) and 8.2% (4 of 49)), all of which were mild or moderate and not serious. Changes from baseline in the UPDRS part III score were minimal in all of the groups (−0.9 ± 0.9, −1.7 ± 0.9 and 0.4 ± 0.9 points (mean ± SE), respectively) without a significant difference between either of the active groups and the placebo group (5 mg: *P* = 0.525, 10 mg: *P* = 0.306).

The incidence of psychiatric events was similar between the 5 mg group and the placebo group, and the incidence in the 10 mg group was lower than that in the placebo group (10.9% (5 of 46), 12.8% (6 of 47) and 4.1% (2 of 49)). The incidence of individual psychiatric events was <5% in each group. Five severe psychiatric events were reported in two patients in the 5 mg group: visual hallucinations, insomnia, paranoia, agitation and irritability, all of which were judged to be related to the treatment.

The incidence of arrhythmic events was similar among the groups (4.3% (2 of 46), 4.3% (2 of 47) and 6.1% (3 of 49)). Each event was reported by only one patient, and the events were of mild to moderate severity.

For vital signs, blood pressure, pulse rate and body weight slightly declined in the active groups. AEs related to vital signs were ventricular extrasystoles (*n* = 1) and hypotension (*n* = 1) in the 10 mg group and weight decrease (*n* = 1) in the 5 mg group. All of these AEs were either mild or moderate. No patients reported any abnormal changes in pulse rate. The incidences of abnormal changes in the electrocardiogram were similar among the groups (4.7% (2 of 43), 4.7% (2 of 43) and 6.3% (3 of 48)).

## Discussion

In the primary analysis of the co–primary endpoints (MMSE and NPI-2 scores), predefined superiority over placebo was not confirmed in either the 5 mg or 10 mg group. However, in the evaluation of cognitive function using MMSE score, the difference between the placebo and 10 mg groups was significant, which is consistent with the previous double-blind study [11]. The mean change in the MMSE score in the 10 mg group was 2.2 points, which was almost equal to the score of 2.3 obtained in our previous study [11].

The improvement in the 5 mg group was found to be significant only in the PPS analysis, although it was also found to be significant in all analyses in the previous study [11]. The results of the present study did not replicate our previous finding, which is probably due to a relatively larger number of earlier discontinuations. In the 5 mg group, eight patients (17.0%) discontinued by week 4 when the blood concentrations of 5 mg donepezil reached the steady state, whereas only one patient (3.0%) discontinued in the previous study. The imbalance of discontinuation was not caused by the dose of 5 mg itself, because only one patient in the 10 mg group discontinued by week 4, while taking the same doses as the 5 mg group until week 6.

In two phase III studies in which the efficacy of donepezil in patients with mild to moderate AD was investigated [22,23], a mean change in the MMSE score of 0.24 to 1.35 points with a difference from the change in the placebo groups of 1.02 to 1.36 points was reported. In contrast, in the confirmatory phase of this study and in the previous double-blind study [11] in patients with DLB, the mean change in the MMSE score in the active groups (5 or 10 mg) was 1.4 to 3.4 points with a difference from the placebo groups of 0.8 to 3.8 points, which exceeded the equivalent scores in the two AD studies. Therefore, these results imply that treatment with donepezil for DLB provides greater improvement in cognitive function than for AD, for which donepezil had already been approved, reinforcing the clinical significance of treating DLB with donepezil.

In the phase II study, donepezil clearly showed dose-dependent efficacy against behavioral and neuropsychiatric symptoms [11]. In the present study, however, the placebo group also benefited from improvement in these symptoms, which represents the failure to replicate the findings in the previous study. Which factors affected the unexpected improvement of behavioral and neuropsychiatric symptoms in the placebo group? Two possible reasons are conceivable in terms of the time of the trials: (1) promotion of disease awareness and improved caregiving methodology brought about by quantitatively and qualitatively enriched disease-related information and (2) the emergence of reports on successful psychosocial interventions in behavioral and neuropsychological disorders related to DLB. Psychosocial factors, as well as brain organic and functional factors, have been reported to cause symptoms such as hallucinations in DLB [24]. Anxiety alleviation, accompanied by enhanced disease understanding, advancement in coping skills and promotion of empathic attitudes through disease education and instructions, may relieve symptoms (for example, frequency or severity of hallucinations) [24,25]. Most of the patients and their caregivers likely received disease education and/or caregiving instructions or acquired information on the disease and its care right before or during the study. The education of and information provided for caregivers may also have increased a positive bias, because NPI is an assessment scale implemented through interviews with caregivers. To lessen the placebo effect, a lead-in period when nonpharmacological treatment is administered has been suggested by a study in which investigators evaluated the efficacy of pimavanserin on psychosis in Parkinson’s disease [26]. The results of our present study support our interpretation and the necessity of disease-specific brief psychosocial therapy in the lead-in period in future studies.

In the confirmatory phase, most of the AEs were mild or moderate in severity. The absence of substantial differences in the incidence of AEs or treatment-related AEs, and the existence of fewer reports on AEs that led to discontinuation in the 10 mg group than in the placebo group, suggest tolerability of donepezil in patients with DLB. The incidence of gastrointestinal symptoms, which are typically observed AEs with ChEI administration, did not tend to increase in the active groups. Another expected risk was parkinsonism. Donepezil may possibly induce or exacerbate extrapyramidal symptoms, which are threatening for patients with DLB. Although it is reported with a slightly higher incidence in the 10 mg group, none of these events were serious, and the UPDRS part III score did not represent significant deterioration in each of the active groups. We found no particular concerns about psychiatric symptoms or arrhythmia.

The interpretation of the present results requires taking some points into consideration. First, the number of patients enrolled by each center was generally small (that is, none by 14 of 72 centers and only 1 by 15 of the remaining 58), possibly due to DLB’s characteristic features, including the faster progression, severe psychiatric symptoms and greater caregiver burden when compared to those with AD [4,27-30]. Similar recruitment difficulties impeded the previous phase II trial and a placebo-controlled study of rivastigmine in patients with DLB [31]. This may have caused a flaw in the interrater reliability of the clinical ratings. However, in this trial, a training and certification course was mandatory for the investigators. A second limitation is the short duration of the RCT phase. The period was set to 12 weeks, considering the above-noted disease-specific characteristics and the result of the previous phase II trial and its extension. The long-term efficacy of donepezil was evaluated in the open-label extension phase and is reported in another paper [13]. Third, because a global measure was not used, the influence of donepezil administration on the global clinical status cannot be inferred, despite its clinically important effect on improvement in cognitive function demonstrated through evaluation using the MMSE.

## Conclusions

The predefined superiority of donepezil over the placebo in the co–primary endpoints was not confirmed. However, significant improvement in MMSE score was demonstrated with 10 mg but not 5 mg. Overall, donepezil was well tolerated in patients with DLB. While paying careful attention to gastrointestinal and parkinsonian symptoms, patients with DLB can safely benefit from treatment with donepezil.

## Additional files

Additional file 1:
**Cognitive Fluctuation Inventory (CFI).** This questionnaire was originally developed in Japanese. The English version is not yet validated. Additional file 2:
**List of all institutional review boards.**

## Abbreviations

AD: Alzheimer’s disease
AE: Adverse event
ANCOVA: Analysis of covariance
ChAT: Choline acetyltransferase
ChEI: Cholinesterase inhibitors
DLB: Dementia with Lewy bodies
FAS: Full analysis set
LOCF: Last observation carried forward
MMSE: Mini-Mental State Examination
NPI: Neuropsychiatric Inventory
PPS: Per-protocol set
RCT: Randomized placebo-controlled trial
SD: Standard deviation
SE: Standard error
UPDRS: Unified Parkinson’s Disease Rating Scale
ZBI: Zarit Caregiver Burden Interview
