# Supplementary figures and images for: Donepezil for dementia with Lewy bodies: a randomized, placebo-controlled, confirmatory phase III trial
Source: Alzheimers Res Ther. 2015 Feb 3;7:4. doi: 10.1186/s13195-014-0083-0 (PMC4338565; doi:10.1186/s13195-014-0083-0)

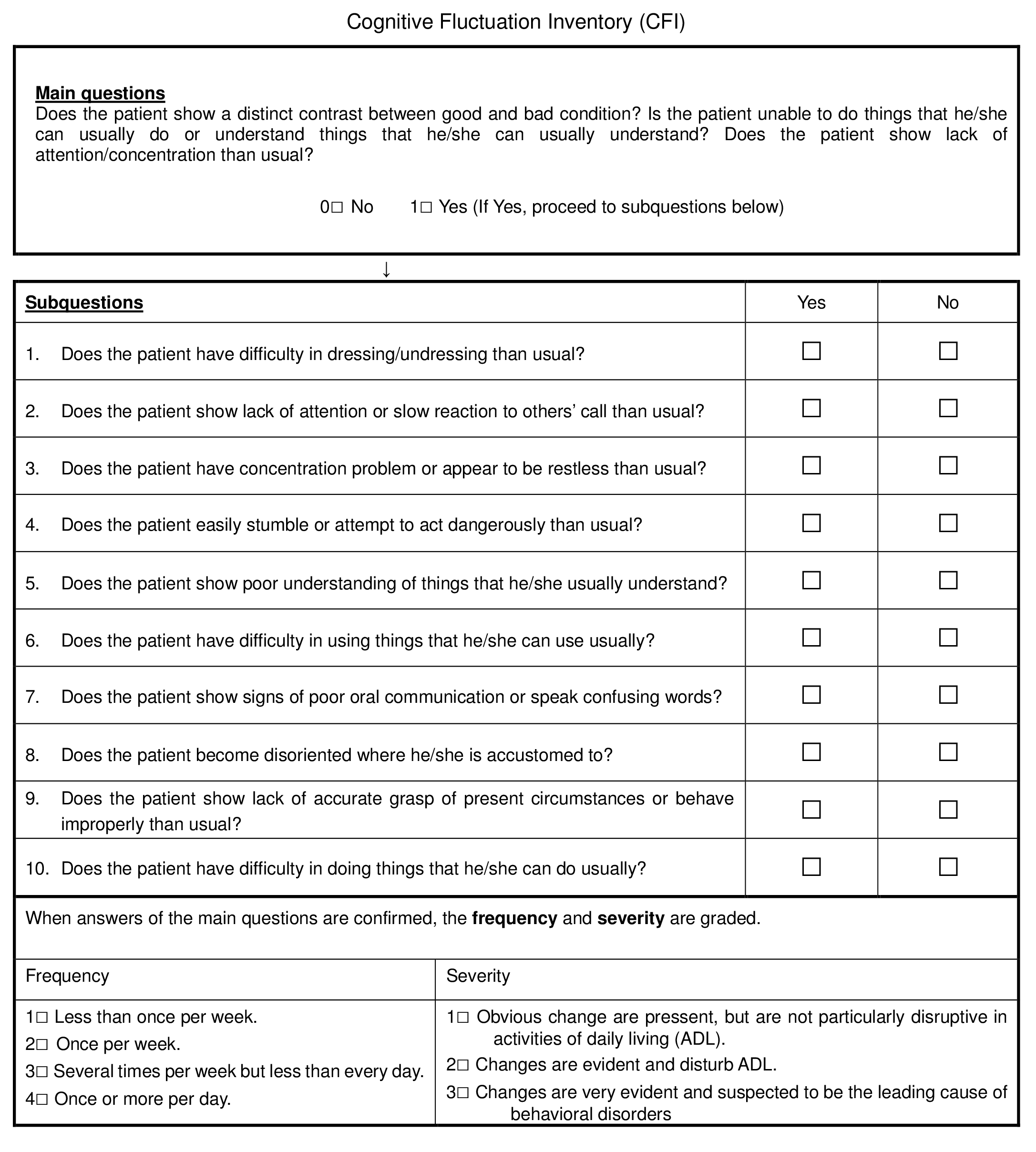

Supplement: Additional file 1: — Cognitive Fluctuation Inventory (CFI). This questionnaire was originally developed in Japanese. The English version is not yet validated. [file 13195_2014_83_MOESM1_ESM.tiff]
